# Deep learning-based system development for black pine bast scale detection

**DOI:** 10.1038/s41598-021-04432-z

**Published:** 2022-01-12

**Authors:** Wonsub Yun, J. Praveen Kumar, Sangjoon Lee, Dong-Soo Kim, Byoung-Kwan Cho

**Affiliations:** 1 Department of Biosystems Machinery Engineering, Chungnam National University, 99 Daehak-ro, Yuseonggu, Daejeon, 34134 Korea; 2 School of Computer Science and Engineering, VIT-AP University, Near Vijayawada, Vijayawada, Andhra Pradesh India; 3 Forest Biomaterials Research Center, National Institute of Forest Science, 672 Jinju-daero, Jinju-si, 52817 Korea; 4 Department of Smart Agriculture Systems, Chungnam National University, 99 Daehak-ro, Yuseong-gu, Daejeon, 34134 Korea

**Keywords:** Imaging and sensing, Computer science

## Abstract

The prevention of the loss of agricultural resources caused by pests is an important issue. Advances are being made in technologies, but current farm management methods and equipment have not yet met the level required for precise pest control, and most rely on manual management by professional workers. Hence, a pest detection system based on deep learning was developed for the automatic pest density measurement. In the proposed system, an image capture device for pheromone traps was developed to solve nonuniform shooting distance and the reflection of the outer vinyl of the trap while capturing the images. Since the black pine bast scale pest is small, pheromone traps are captured as several subimages and they are used for training the deep learning model. Finally, they are integrated by an image stitching algorithm to form an entire trap image. These processes are managed with the developed smartphone application. The deep learning model detects the pests in the image. The experimental results indicate that the model achieves an F1 score of 0.90 and mAP of 94.7% and suggest that a deep learning model based on object detection can be used for quick and automatic detection of pests attracted to pheromone traps.

## Introduction

Pests cause multiple problems in South Korea, especially in the fields of agriculture and forestry. Nilaparvata lugens causes a huge problem in rice farming in South Korea, damaging the rice crop directly by feeding and spreading viruses^1^. There are various pests such as termites, lumberjacks, and beetles that damage wood. Platypus koryoeonsis was reported to be damaging Korean oak trees, infesting the trees by feeding on them^2^. Aphanisticus congener, which is a newly migrated pest reported on Jeju Island, Korea, is causing problems by damaging the leaves of turfgrass leaf^3^. Monocellicampa pruni Wei led to severe losses in Japanese plum farming by damaging the young fruits in the Jeonnam area in 2018 and 2019^4^. Thus, pest control is necessary to protect forests and crops nationwide.

The black pine bast scale, the pest dealt with in our experiment, is one of the most serious pests in Korea. The black pine bast scale hibernates during the summer; from December to February, the larvae damage the trees by sucking the sap. Pine trees that have been damaged by pine bark insects have brown spots on their skin, and when the density of pests is high, this causes extreme weakening of the trees. When the leaves of the lower branches of the crowns turn brown, the growth of these trees decreases, and the number of cells per unit area decreases, resulting in the death of branches or trees^5^.

There have been multiple approaches to pest detection such as computer vision and machine learning-based approaches, deep learning-based approaches, and real-time application development using these approaches. The Conventional approach like observation with the naked eye will not be much effective since the pest identification depends on the individual’s pest identification skills. A few of the computer vision and machine learning-based approaches are as follows. Silveira and Monteiro used support vector machine with image features and 1D Hough Transform to detect and measure the butterfly eyespot patterns^6^. A detection and segmentation algorithm^7^ using GMM and DRLSE have been developed for the automatic monitoring of Lepidopteran pest species. Bodhe and Mukherji^8^ proposed a whitefly detecting algorithm using color-based image segmentation techniques. A vision-based pest detection method^9^ was developed to detect the parasites found on strawberry plants based on SVM (Support Vector Machine) classification. Kirkeby et al.^10^ proposed a method to identify the flying insects automatically using optical sensors and machine learning. Rapid and low-cost insect detection method^11^ using the bag of visual words (BoVW) algorithm was proposed to analyze the trapped species. An machine learning-based whitefly classifier^12^ was developed using noise removal, contrast enhancement, k-means, Gray Level Run Length Matrix (GLRLM), Gray Level Co-occurrence Matrix (GLCM), SVM, and a Bayesian classifier. The real-time applications were also developed by the researchers. The authors in the paper^13^ developed a prototype using Raspberry Pi to send pest images to the server for automatic monitoring of sucking insects. Liu et al.^14^ developed a real-time computer vision based robot automobile monitoring system using inverse histogram mapping and object contour to recognize Pyralidae.

Recently, much research has been carried out using deep learning techniques to detect the pests. Artificial neural network (ANN) based model^15^ was applied to detect the butterfly species using color and texture features. Generally, classification and detection methods with CNN have been used in recent pest management research. The CNN based models are trending for their accuracy and efficiency. A CNN (convolutional neural network) model^16^ that uses the GAN image augmentation technique was introduced for insect pest classification enhancement. Multiple algorithms^17^ were trained to classify moths using features like shape, color, and texture. A smartphone camera was used to take the images of codling moths, then the researchers trained a network^18^ based on the captured images. An improved deep learning pipeline^19^ was proposed to count the agricultural crop pests where the CNN algorithm for the automatic localization and counting of agricultural crop pests was trained using RPN and NMS techniques. Ferentinos^20^ proposed deep learning models for plant disease detection and diagnosis. The plant pest detection was evaluated using an artificial nose system^21^. Selvaraj et al.^22^ developed AI-powered banana diseases and pest detection method based on deep convolutional neural networks (DCNN). Ding and Taylor^23^ introduced an automatic detection pipeline to detect codling moths using convnet architecture. A pest and crop disease classification model^24^ was developed using VGG (Visual Geometry Group). Sun et al. developed a deep learning detection method^25^ using ResNet and MobileNet to detect the red turpentine beetle. Furthermore, the trained weights were adopted to Nvidia Jetson TX2 and Raspberry Pi3, showing the possibility of remote detection on mobile platforms. The detection models^26^ for multiple pests and diseases in a single image was developed using Inception and ResNet architecture. Liu et al. used RPN and CNN architecture for large-scale multiclass pest detection and classification^27^. Anchor-free region convolutional neural network^28^ was employed via an end-to-end way for multiple categories of agricultural pest detection. Liu et al.^29^ used the hybrid global and local activated features to develop deep learning models for automatic multiclass wild pest monitoring. Bio-inspired method^30^ was proposed to detect and recognize insect pests.

The drawbacks of existing traditional pest detection methods are (1) lack of suitability for mobile devices, (2) deficiency of robustness, (3) lower accuracy, and (4) a high equipment cost. Hence, there is a need for new pest detection methods. The authors in papers^31,32^ studied that the pest density caught by a pheromone traps and the pest densities on crops are correlated. These studies paved a way for image-based pest detection methods. To facilitate the image-based pest detection methods Scoutbox system has been introduced by Agrocares for taking photos^11^. But these systems are expensive and heavy to carry because of high end camera and protection box. To overcome these drawbacks, a smartphone-based application for pest detection is developed. The developed smartphone application uses our trained model for rapid detection of pest traps and ensures the easy accessibility. An imaging system was developed to aid in image acquisition. The recent object detection models were evaluated for pest detection and the best model is deployed on the server environment. Additionally, it is aided by the smartphone application for practical usage.

## Materials

### Image acquisition

In our experiment, a Huawei P30 Pro model smartphone, which had a high pixel counting image sensor, was selected as the camera equipment for the sticky trap image capturing system. The advantages of this equipment were the ease of use in the field and the high accessibility. Moreover, the selected camera equipment can be switched between macro lenses, general lenses, and wide-angle lenses on a 40-megapixel imaging sensor. Additionally, data access, application creation, and utilization can be done with ease. To develop a total pest identification and counting system using a combination of a general smartphone and an additional lens to improve the field applicability, the built-in lens method was selected since it gave the best imaging results.

A frame was needed to ensure the stable movement of camera equipment and the pheromone trap. The size of the overall frame was 360 mm × 460 mm. To maintain the clarity of the image, the entire trap was divided into subimages with an overlapping area of 30% between neighboring subimages. Two CNC linear rail belt slides were also adopted for smooth movement of both the camera and the pheromone trap. Arduino was adopted to control the motors and trigger the smartphone camera. A CNC linear belt slide was connected with the frame support for stable use. Then, a slider system was connected to the exoskeleton for imaging system adoption. A motor–LED connecting bracket was specially designed and 3D printed to connect the LED and motor. Additionally, a bracket slot was drilled for the LED adjustment.

Vinyl covers were applied to the trap for the protection and preservation of the pests. However, the vinyl cover caused light reflection and resulted in the dispersion of the entire image. As a solution, a dome-shaped LED was adopted. The dome-shaped cover was irradiated with LED light, and the light reflected from the cover was indirectly irradiated to the target object. The dome-shaped LED light prevents shadowing of irregularities and halation of the glossy surface. Additionally, the dome-shaped LED facilitates the smooth diffused light that could be irradiated evenly on an irregular target object, making the surface uniform, and making the contrast to the detection point clear.

A 10 k digital potentiometer MCP 4131 was used to trigger the camera since the electrical current should be passed only when the camera is operated. Then, the Bluetooth imaging remote was connected to the digital potentiometer. When a signal at a high state was sent from Arduino to the digital potentiometer, the circuit got connected and the signal was delivered to the pin of the Bluetooth imaging remote. The signal from the pin triggered the smartphone to capture an image when the camera application was running. In this way, the phone camera could be used whenever the signal was sent.

The motor driver DRV-8825 was used to control the motor. The driver was powered with 5 V from Arduino and a signal from Arduino was sent to the driver. When the signal from Arduino was high, an internal circuit was opened and power from the 24 V power supply could enter the driver. Depending on the signal, the motor could be moved clockwise or counterclockwise; also, the overall speed of the motor could be controlled.

Two buttons were added to the circuit for controlling the system. Since Huawei’s 40 MP wide-angle camera uses a 9:16 ratio sensor, initially, the halation and the background should be cropped. Then, the coordinates of the yellow sticky traps inside the original image were obtained. Based on the obtained coordinates, the subimages were cropped (capturing subimages from the entire pheromone trap image) as follows.

When the first button was pressed, the two motors individually moved the imaging target and the camera to the desired point. Then, the first subimage was captured. The camera equipment and the pheromone trap were moved to the next desired position and the second subimage was captured with a 30% overlapping area^33^ between the first and second sub-images. This process was continued until all 16 subimages were captured over the entire image, with a 30% overlapping area between the neighbors. This was controlled by our program, written on Arduino. Due to the 0.1 mm precision of the motor, the coordinates of each point mentioned above would mismatch. To solve this problem, a second button was added. When the second button was pressed, the carrier on the rail could travel until it reached the B05 end-stop switch. The B05 end-stop switch was used as a starting reference point of the system and was used to avoid the abovementioned mismatch.

**Figure 1:**
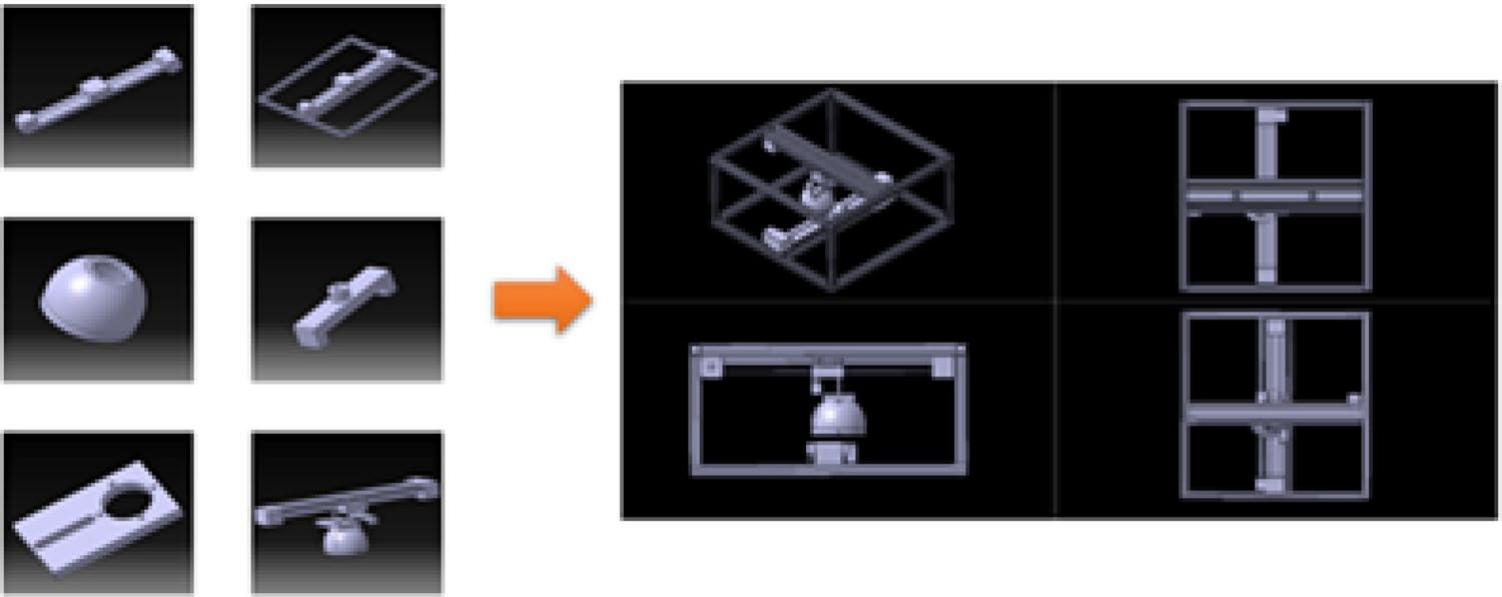
Imaging system and schematics of its parts.

**Figure 2:**
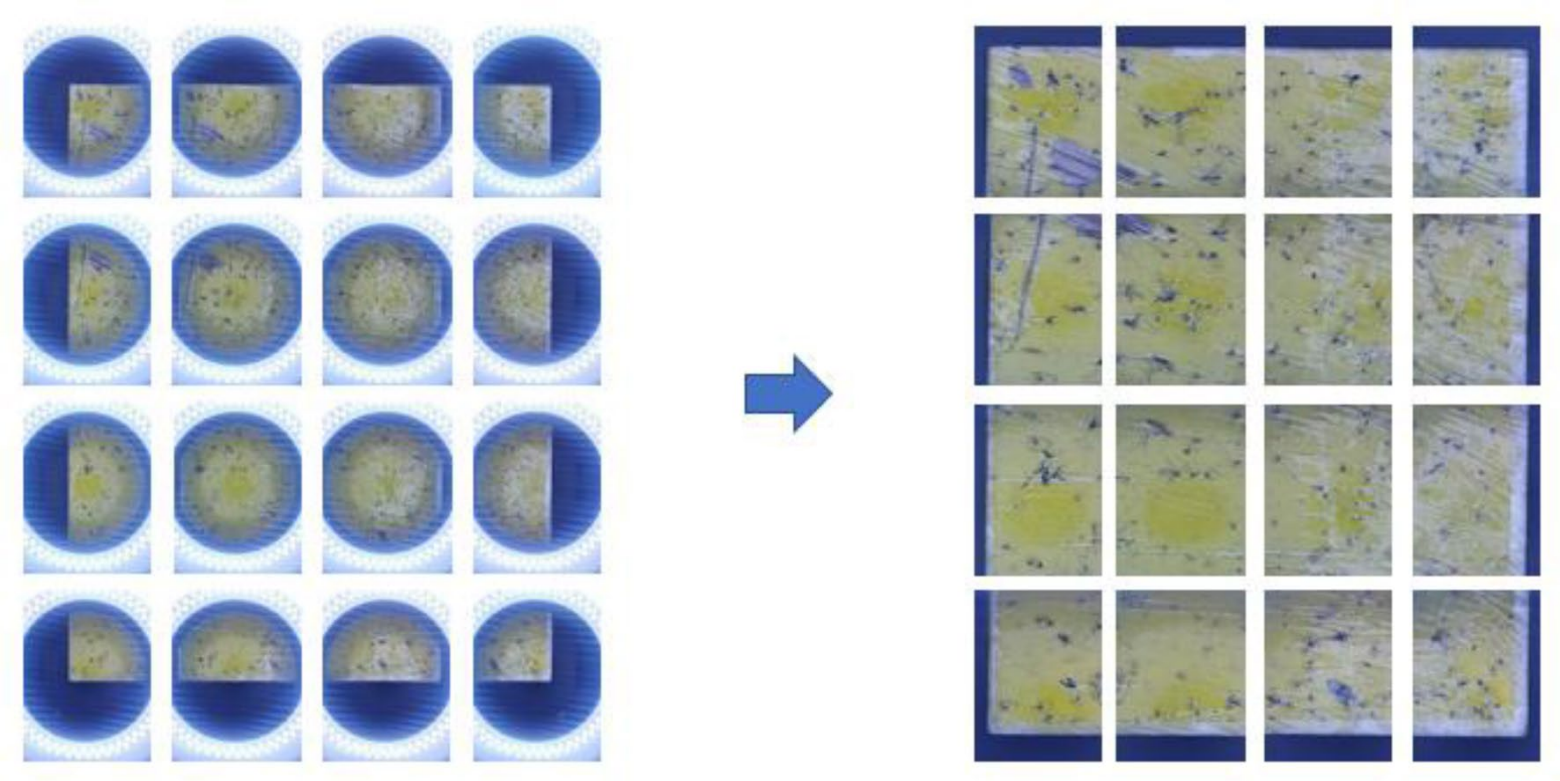
Image cropping demonstration.

Using this mechanism, the motor could return to its starting reference point after each task was finished or the task was disturbed. The schematics of the imaging system are shown in Fig. 1 and the image cropping is shown in Fig. 2 and Supplementary Fig. 1. All the captured subimages were used for training the pest detection model. Finally, the subimages for each pheromone trap were stitched into a whole image. The image stitching process is explained in the Image Stitching section.

### Dataset description

In the experiment, 400 pheromone traps were used and images of the black pine bast scale were taken and labeled according to their characteristics. The number of pheromone traps used for the training and testing datasets was 280 and 120, respectively. Using Python’s COCO annotator library, each of the pests in the images was labeled. While labeling was performed, care was taken that the labeling bounding box tightly covered the pests and had a greater influence on accuracy improvement. The labeling results were in the format of json files reflecting the x and y coordinates of the bounding box. In the experiment, the training dataset consisted of 4480 subimages and the testing dataset consisted of 1920 subimages. A total of 2826 target pests and 1308 target pests were in the pheromone traps used in the training and testing datasets, respectively. The details are given in Table 1. To validate the pest detection models, 30% of the training dataset images were chosen.

**Table 1:** Dataset details.

Pest name	Training		Testing	
	#traps	#target pests	#traps	#target pests
*Matsucoccus thunbergianae*	280	2826	120	1308

## Methods

The experiments done in this study are based on the Pytorch library and run on three NVIDIA QUADRO RTX 4000 GPUs under CUDA 10.1 and cuDNN 7.6.5 graphic drivers. Two INTEL XEON GOLD 6230 Processor CPUs with a RAM size of 256 GB were used to assist the GPU’s performance. The specifications of the deep learning workstation are listed in Supplementary Table 1. Both the minimum and maximum batch sizes were tested on the workstation. In this section, deep learning models, image stitching, and smartphone application development for pest detection are discussed.

### Model

Deep learning models can learn the features directly by themselves and detect the features that humans do not understand. However, the machine learning models need to be trained to learn these features. In this experiment, transfer learning models are used due to their advantages such as ease of training, lower training time, and better performance of neural networks. The pretrained models used in our experiment are YOLO v3^34^, YOLO v4^35^, and YOLO v5^36^.

The YOLO V3 model uses the Darknet-53 architecture. It is a 106-layered network that has 53 layers trained on Imagenet, used as a backbone, and 53 layers for the detection task. It predicts bounding boxes using feature maps with various sizes of resolution and, when predicting a class, binary classification using sigmoid for each class is applied. This architecture stacks blocks of 3 × 3 convolution and 1 × 1 convolution in succession. Instead of max pooling, the convolution stride is taken as 2 to reduce the resolution of the feature map. In addition, residual values are transmitted using a skip connection. After passing through average pooling and a fully connected layer in the last layer, the classification results are given as output through Softmax.

To solve the problems with previous YOLO models that were not efficient for detecting small objects, the YOLO v4 model was developed. It solved the problem by increasing the input resolution of the model. In addition, the number of layers was increased to enhance the receptive field. Since high expressive power is required for the simultaneous detection of objects of various types and sizes in one image, the number of parameters has been increased. The model uses CSPNet^37^-based CSPDarkNet53 as the backbone, which proposes a Cross Stage Partial Network structure that can mitigate the heavy inference cost and minimize the loss of accuracy. Based on this method, the inference cost and memory cost could be reduced. In addition, it is proposed that the loss of accuracy is small since it has a positive effect on training by dividing a gradient flow.

**Figure 3:**
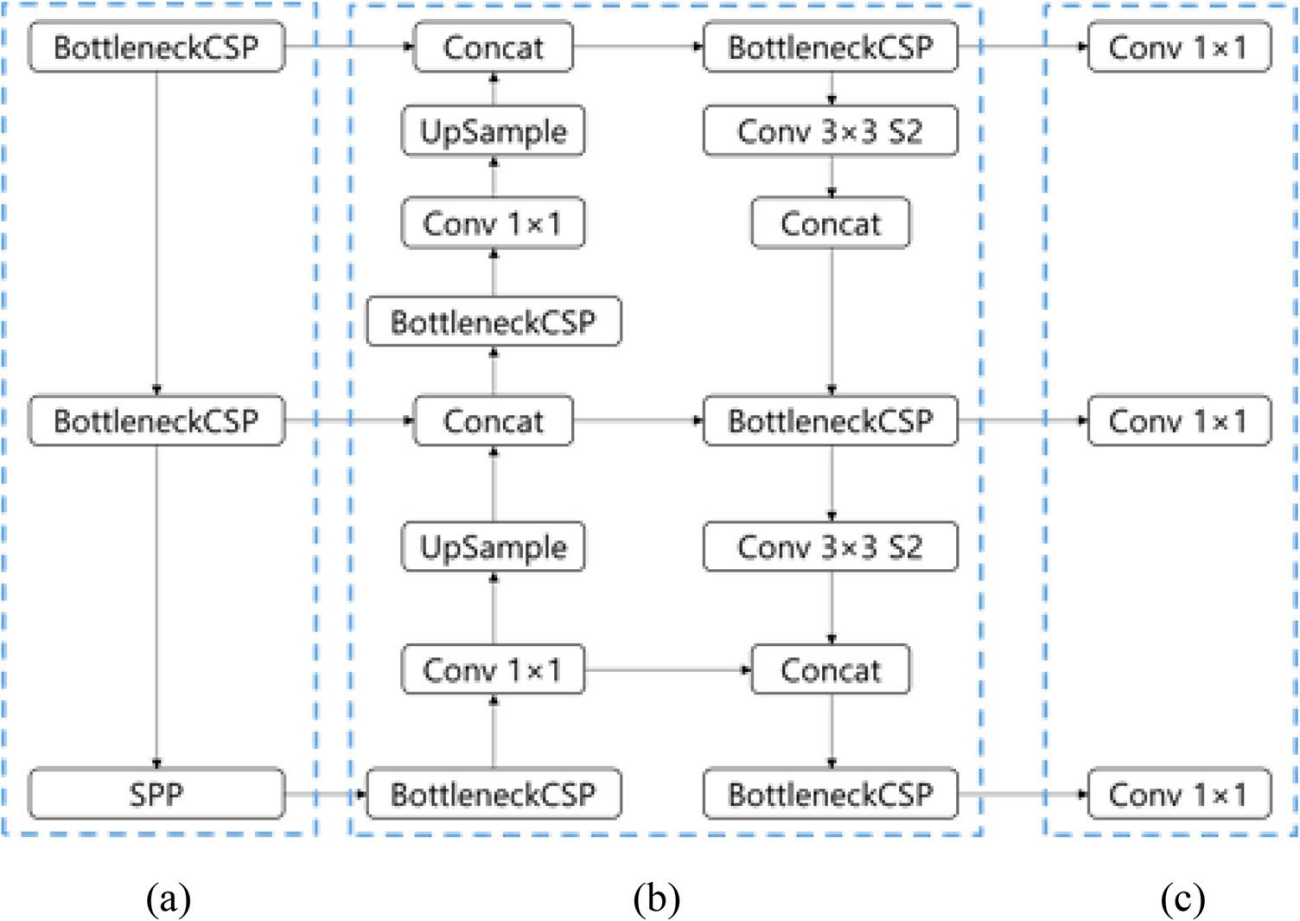
YOLO v5 architecture. (**a**) CSPDarkNet backbone. (**b**) PANet neck. (**c**) YOLO head.

While the conventional YOLO v3 has a high number of frames per second (FPS), the mean average precision is relatively low. However, YOLO v5 has higher performance in terms of both FPS and mAP. The YOLO v5 models are divided by size, unlike other YOLO models. The criterion for dividing this is the difference between the model depth multiple and the layer width multiple. Accuracy and speed are generally incompatible, so it is challenging to achieve both at the same time. YOLO v5 s, being the fastest model, has decreased accuracy, while the YOLO v5 x model performs slowly, so the accuracy improves. YOLO v5 also uses CSPNet-based CSPDarkNet as the backbone for feature extraction. Based on the features acquired from the backbone, the head of the model performs the detection task. This model initially sets the anchor box and uses it to create the final bounding box. Like YOLO v3, it creates bounding boxes at three scales and three anchor boxes are used at each scale. The YOLO v5 architecture used in our experiment is shown in Fig. 3. The training loss of the various YOLO models in our experiment is shown in Supplementary Fig. 2.

In addition to model selection, model optimization was performed. Deep learning model optimization was performed by adjusting the hyperparameters that control the general behavior of the model. The three widely adopted methods^38^ for hyperparameter optimization are: (1) manual search, (2) grid searches, and (3) arbitrary search. Although there have been several attempts^39,40^ to understand and visualize what is happening in deep learning networks, the empirical approach^41^ is still one of the effective approaches to optimizing hyperparameters.

To train our models for detecting the black pine bast scale, the SGD (stochastic gradient descent) optimizer was used along with a learning rate and momentum. To find the optimal hyperparameter values, the Hyperparameter Evolution library^43,44^ is used in our experiment. The batch size relied on the computing ability and available memory. Once the models were trained, the best-performing model was deployed to our lab server. After the pests were detected using the model, the subimages were stitched by an image stitching method, as described in the following subsection.

### Image stitching

To maintain the clarity of the image, each test image of pheromone traps was divided into 16 subimages. To observe the inference results together and count the total number of pests within the trap, a stitching method must be applied. The stitching method^33^ was implemented with OpenCV-Python. The size of the stitched image was about 3456 × 4608 pixels.

The image stitching needs annotations for matching the features of two images. The images are imported and converted into grayscale images. The SIFT (Scale-Invariant Feature Transform) method was used to extract the key points and sift descriptors. The image was annotated with features detected by SIFT. Once the descriptors and key points were extracted, the correspondences between the images needed to be found. The overlapping points give the orientation of the image according to the other image. And based on the common points, it can be found if the second image is bigger or smaller or has it been rotated and then overlapped, or maybe scaled down/up and then fitted. All such information is yielded by establishing the correspondences.

To match the features of the images, the FLANN (Fast Library for Approximate Nearest Neighborhood) method, which contains a collection of algorithms optimized for fast nearest-neighbor search in large datasets and high-dimensional features, was used. Often in images, there are chances that the features may exist in many places in the images. Therefore, the matches are filtered out to obtain the best matches. With the matched features, the perspectives of the images are warped using a homography matrix and then cropped to filter out the overlapping area. Finally, the images are stitched together. This process of the image stitching algorithm is shown in Supplementary Fig. 3.

### Application

The application was configured as follows so that the data could be freely used as input/output. Jupyter notebook was used for the application development. With aid from hybrid web app platform, accessing the lab server was possible via a smartphone. The images obtained from the smartphone could be transmitted to the deep learning workstation, and then to the lab server. The deployed model in the lab server was used to detect the black pine bast scale in the images. The bounding box and classification accuracy were displayed on the same image after black pine bast scale detection. The process of the smartphone application was represented in Supplementary Fig. 4.

**Figure 4:**
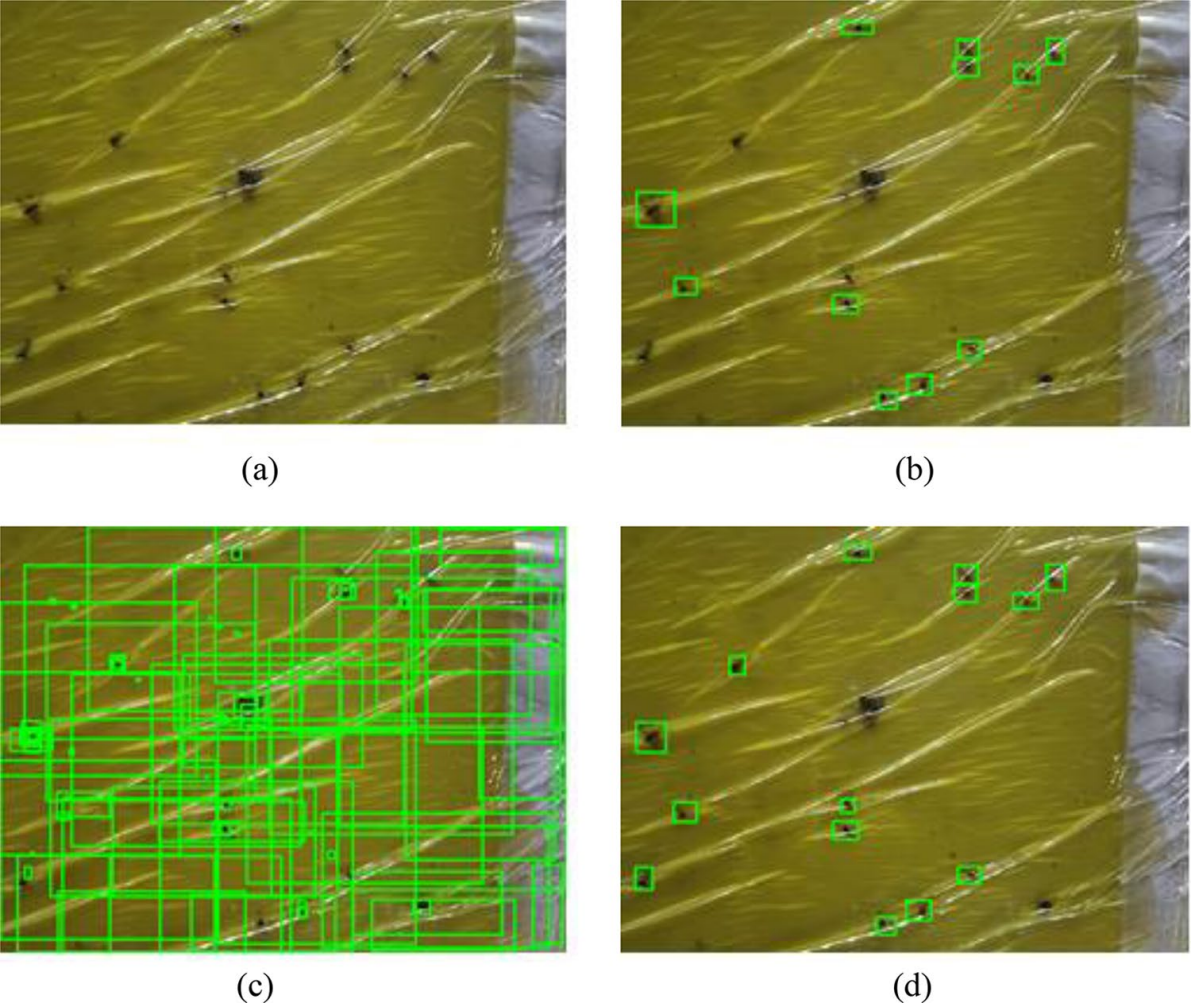
Pest Detection by the deep learning model. (**a**) Original image. (**b**) Ground truth labels. (**c**) Initial candidates predicted with network. (**d**) Refined candidates.

### Experiment settings

Through the visualization of the trained model, rough validation of the model is possible. As the training proceeds, the model proposes many anchors, represented as target candidates. Further visualization can be achieved by extracting the weights of each convolutional layer.

In our experiment, the number of epoch was set to 244. The learning rate, momentum and decay were set to 0.0097, 0.9338 and 0.00025 respectively. The values of these hyper parameters were optimized using Hyperparameter Evolution library^43,44^. The model performance was validated with validation datasets. The training time of the best performing model took 2.85 h with a deep learning workstation with the configuration given in Supplementary Table 1. The training durations of the various YOLO models are given in Supplementary Table 2. In our experiment, the batch size was set to 3. As shown in Supplementary Fig. 2, training loss can continue to decrease. This shows that the model can learn how to detect the properties of pests more efficiently at the beginning of the training phase.

**Table 2:** Performance comparison with other models.

Model	Precision	Recall	F_1_ score	mAP (%)
Fast RCNN	0.89	0.65	0.75	89.6
Faster RCNN	0.91	0.67	0.77	90.1
RetinaNet	0.90	0.69	0.78	89.8
YOLO v5l	0.88	0.92	0.90	94.7

### Ethical approval

This article does not contain any studies with human participants or animals (vertebrates) performed by any of the authors.

## Results

### Performance Measures

In the experiment, performance measures such as precision, recall, F_1_ score, and mean average precision (mAP), as given in the following equations, were used for evaluating the pest detection model.

1 $$precision\;(P) = \frac{True\;Positive}{{True\;Positive + False\;Positive}}$$

2 $$recall\;(R) = \frac{True\;Positive}{{True\;Positive + False\;Negative}}$$

3 $$F_{1} \;score = 2 \times \frac{P \times R}{{P + R}}$$

4 $$mAP = \frac{1}{n(T)}\sum\limits_{r \in T} {AP_{r} }$$

where true positive is the number of correctly identified pests, false positive is the number of falsely identified pests, false negative is the number of unidentified pests, T is the sequence of threshold values, n(T) is the number of threshold values, and AP_r_ is the maximum precision of all recall values greater than r.

If the precision, recall, F1 score, and mAP are closer to 1, the trained model shows higher detection accuracy; if their values are closer to 0, the trained model shows lower detection accuracy.

### Model

The trained models performed the pest detection task as intended, as shown in Fig. 4. Even though the developed methods function well, there were a few problems, such as the multiple detection boxes around the ground truth label, as shown in Fig. 4c. To solve this problem, an IOU-based thresholding algorithm was used. The detection method was also updated to show the target class and its classification accuracy. There was a problem with multiple detections on a single label. To overcome this, the threshold technique was applied. Generally, a threshold for IoU of ≥ 0.5 will give better object detection results. In our experiment, we evaluated the pest detection with IoU threshold of 0.5 for all the models. With this thresholding technique, the problem of multiple detections on a single label was solved.

**Figure 5:**
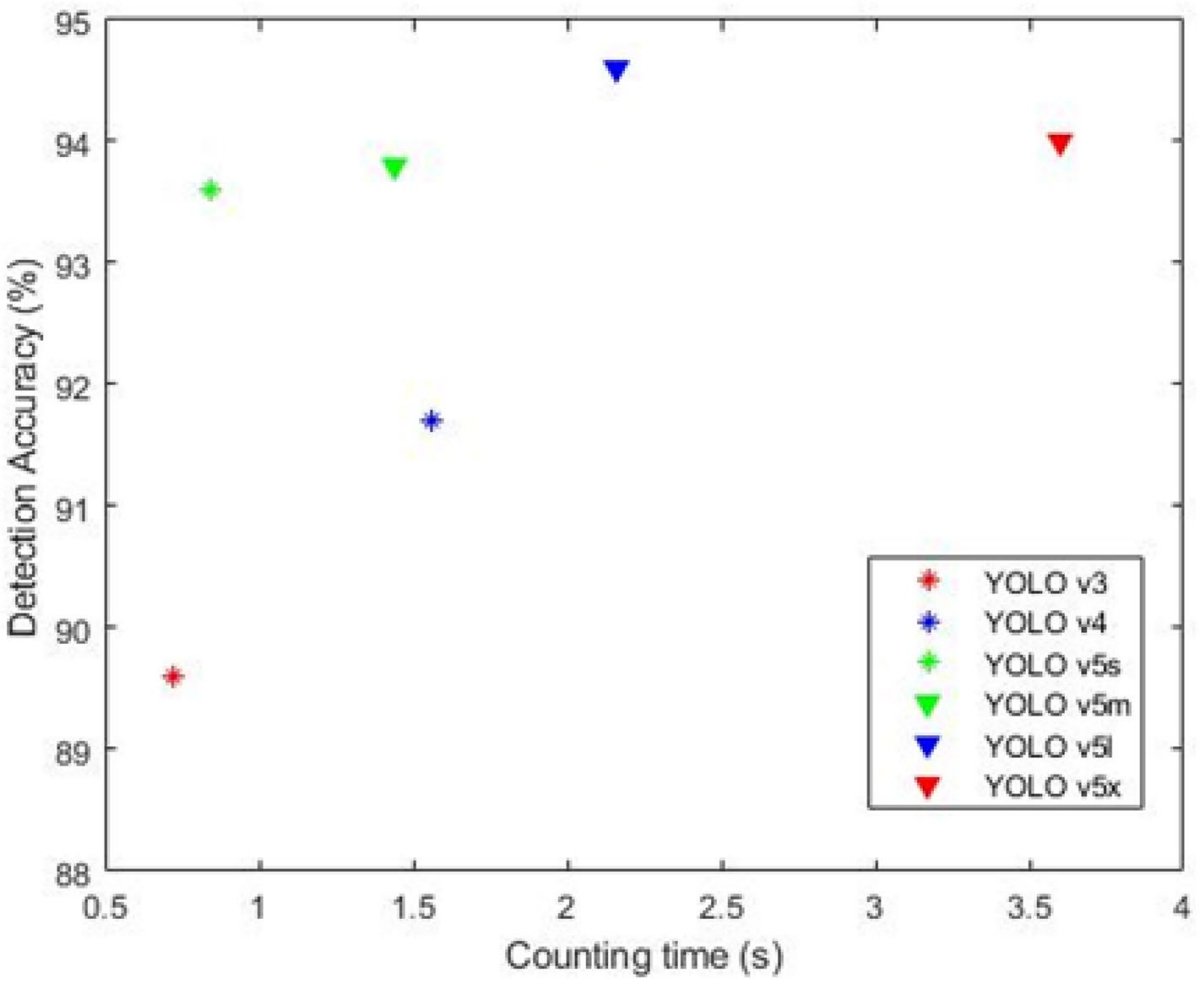
Detection Accuracy-Counting time graph of various YOLO detection models for entire dataset.

Figure 5 shows the detection accuracy-counting time graph of various YOLO models in pest detection. Among these models, the YOLO v5l model achieved the highest mAP (Detection Accuracy) value of 94.7%. Hence, this model was used to compare with other pest detection models such as Fast-RCNN, Faster-RCNN and RetinaNet. The frameworks of the other models are given in Supplementary Fig. 5. The Fast-RCNN algorithm uses Selective Search which is a greedy algorithm and hence they don’t produce best result^45^. Faster-RCNN consists of Deep fully convolutional network, Region Proposal Network (RPN), ROI pooling, Fully connected networks, Bounding box regressor and Classifier. Faster-RCNN use RPN instead of Selective Search and the number of times it runs RPN is less than that of the number of times when compared with Selective Search^46^. The number of anchors are obtained by Non-Maximum Suppression^46^ and thus O(N^2^/2) will be the complexity^47^. The ROI has one pyramid layer and hence O(1) is the complexity^47^. The YOLO models use K-means clustering to find the boxes in training data and hence O(N^kd^) is the complexity^47^ will be where k is the number of images and d is the dimension of images. The YOLO model creators made the algorithm as fastest among the models that are compared in this paper by using thorough and stable optimization techniques^47^. The model size, training time and inference time of various pest detection models are given in Supplementary Table 3. The performance comparison of the YOLO v5l model with other pest detection models is shown in Table 2. It is observed from Table 2 that the YOLO v5l model performed better than the other models for detecting the black pine bast scale pest. The YOLO v5l model achieved precision and recall values of 0.88 and 0.92, respectively. The high recall value shows that the number of pests unidentified was lower compared with the other models. Additionally, it achieved the highest mAP value of 94.7% and a F1 score of 0.9, which are higher compared with the other models given in Table 2. The performance of the YOLO v5l model is also visually compared with machine learning based pest detection methods and is shown in Supplementary Fig. 6.

It is observed from the Supplementary Fig. 6 that the YOLO model performs better than the machine learning models. The machine learning models identify the most of the other insects as black pine bast scale which reduce the pest detection accuracy. Also, some of the background regions are identified as pests. Whereas, the YOLO v5l model detects the black pine bast scale pest more accurately. These indicate that the YOLO v5l model is capable of identifying the black pine bast scale pest better than the other models. Hence, the YOLO v5l model was deployed in our server. There also occur a few limitations such as multiple detection and missed detection with this model and is given in Supplementary Fig. 7. These limitations may occur when the insects are too close to each other or overlapping each other (i.e.) the position of insects in the pheromone traps and require further study in the model development.

### Smartphone application

The Hybrid Web App platform was remotely connected the user’s smartphone with the lab server’s Jupyter Notebook environment. The simple UI was composed of an image viewer, an uploader, a run button, and a text encoder that showed the count results. To use the application, the user must download the apk file and install it on a smartphone. After the installation is done, the user can enter the application and press the button that directs the user to the hybrid web application domain. The user then presses the upload button, which directs the user to upload the images. Then, the pests are detected based on the deployed model. Accordingly, it shows the black pine bast scale detection results. The framework of the application is represented in Supplementary Fig. 8.

## Conclusion

In this study, various methods were used to effectively detect the black pine bast scale from the captured image data on pheromone traps. With the image data, various object detection deep learning methods were investigated. Various models were analyzed to detect the black pine bast scale. The overall performance and execution time showed that the developed models have the potential to accurately and rapidly detect individual pests within the pheromone trap. Among these deep learning models, the YOLO v5l model had the highest F1 score of 0.90 and mAP of 94.7%. Hence, this model was deployed in our lab server. However, further studies are needed to improve the model’s accuracy and inferencing speed. In addition, the image stitching approach was used, and a new smartphone application was developed for efficient and convenient analysis.

## Supplementary Information

Supplementary Information.

## Data Availability

The code used to identify the examined insect species is not freely available.
