# Supplementary material for: Deep learning-based system development for black pine bast scale detection
Source: Sci Rep. 2022 Jan 12;12:606. doi: 10.1038/s41598-021-04432-z (PMC8755754; doi:10.1038/s41598-021-04432-z)
Supplement: Supplementary file 1 — Supplementary Information. [file 41598_2021_4432_MOESM1_ESM.docx]

Deep learning-based system development for Black Pine Bast Scale Detection

Wonsub Yun^1,⸸^, J. Praveen Kumar ^1,2,⸸^, Sangjoon Lee ^1^, Dong-Soo Kim ^3^, and Byoung-Kwan Cho ^1,4,*^

^1^ Department of Biosystems Machinery Engineering, Chungnam National University, 99 Daehak-ro, Yuseonggu,Daejeon 34134, Korea
^2^ School of Computer Science and Engineering, VIT-AP University, Near Vijayawada, Andhra Pradesh, India
^3^ Forest Biomaterials Research Center, National Institute of Forest Science, 672 Jinju-daero, Jinju-si 52817, Korea

^4^ Department of Smart Agriculture Systems, Chungnam National University, 99 Daehak-ro, Yuseong-gu, Daejeon 34134, Korea

^*^corresponding: chobk@cnu.ac.kr
^⸸^These authors contributed equally to this work


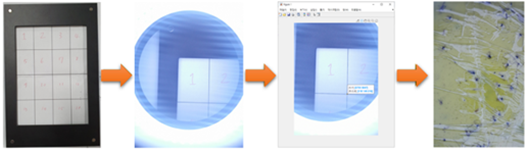


**Suplementary Figure 1.** Image cropping mechanism.


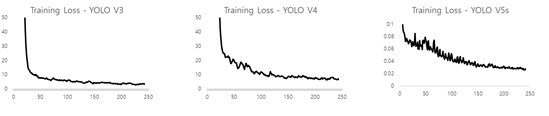


(a) (b) (c)


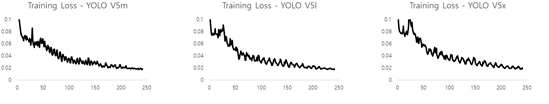


(d) (e) (f)

**Suplementary Figure 2.** Training loss of six different YOLO models in our experiment. (a) YOLO v3. (b) YOLO v4. (c) YOLO v5s. (d) YOLO v5m. (e) YOLO v5l. (f) YOLO v5x.


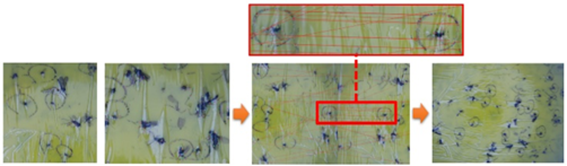


(a) (b) (c)

**Suplementary Figure 3**. Image stitching algorithm. (a) Keypoints extraction. (b) Keypoints matching. (c) Stitched Image.


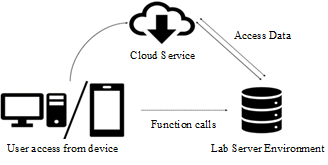


**Suplementary Figure 4.** Mechanism of hybrid web app platform.


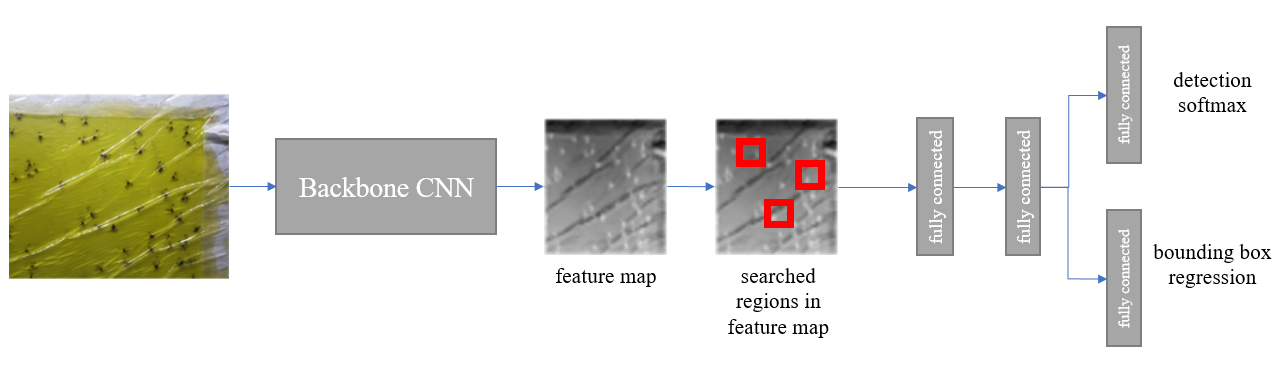


(a)


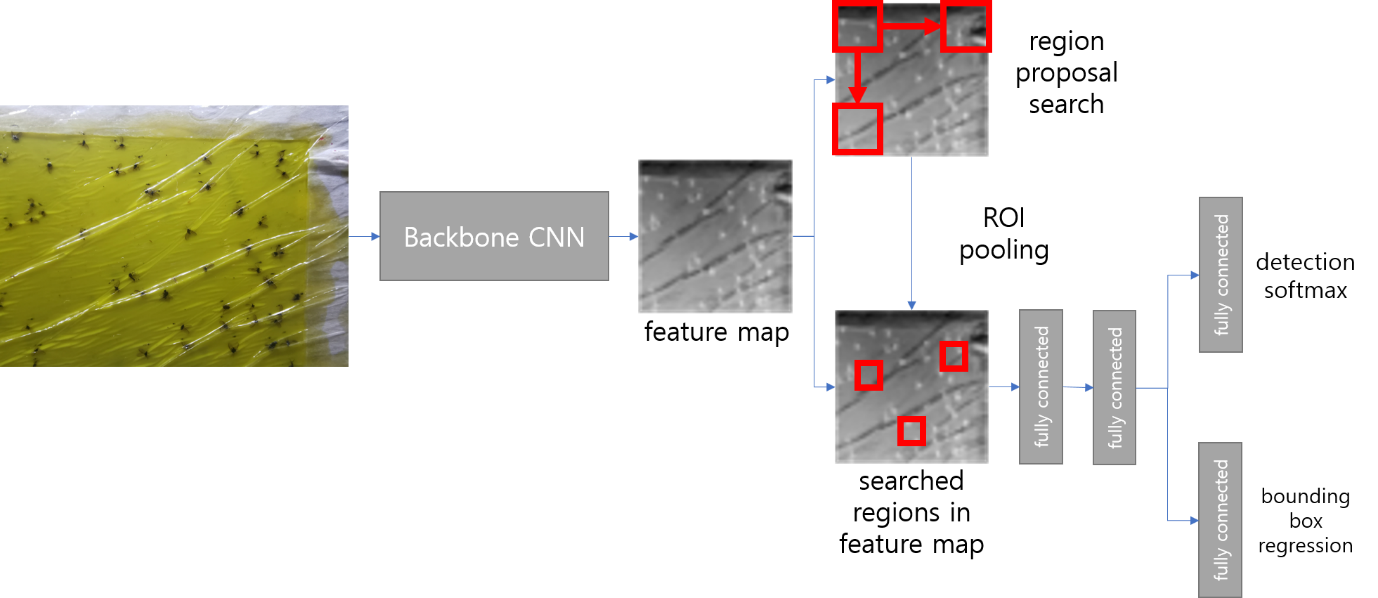


(b)


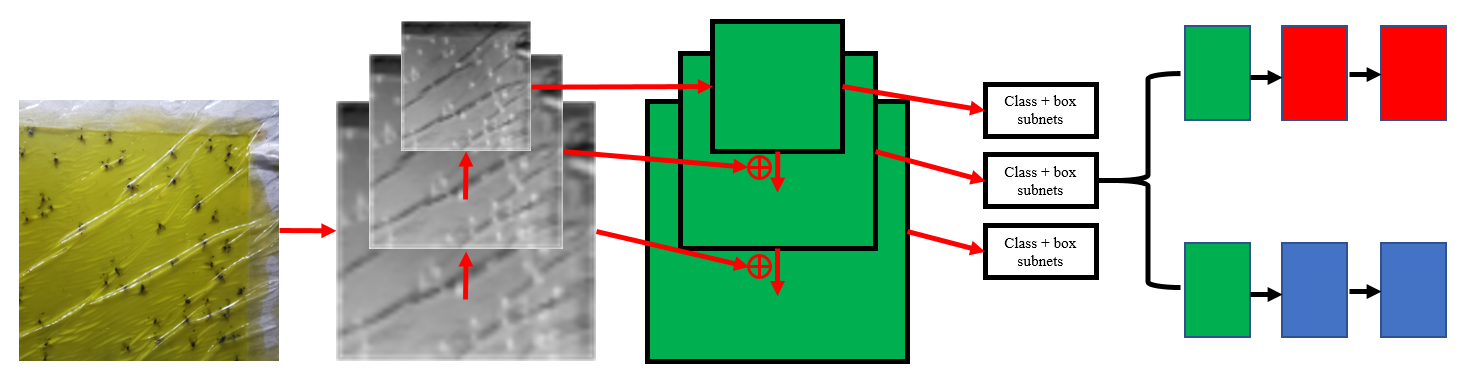


(c)

**Suplementary Figure 5**. Pest detection model framework. (a) Fast-RCNN. (b) Faster-RCNN. (c) RetinaNet.


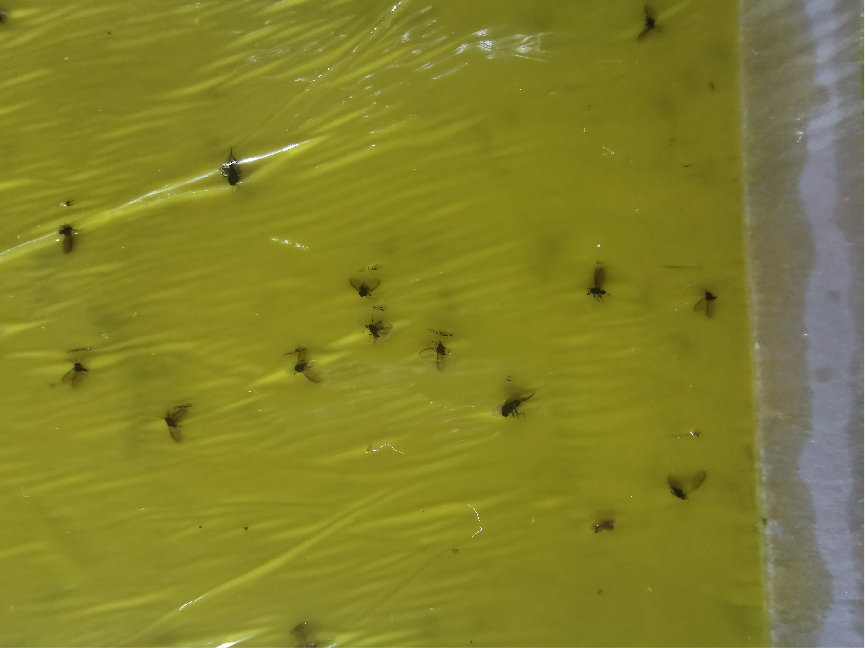

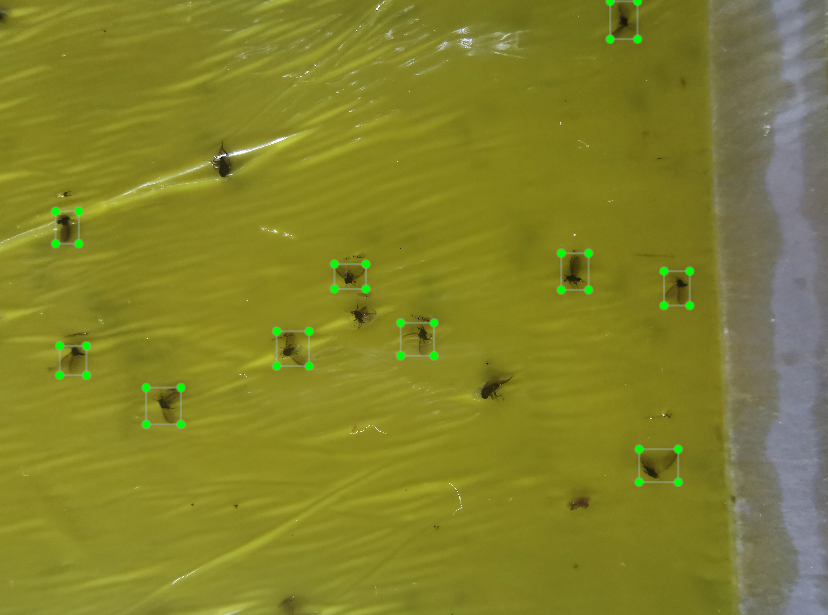


1. (b)


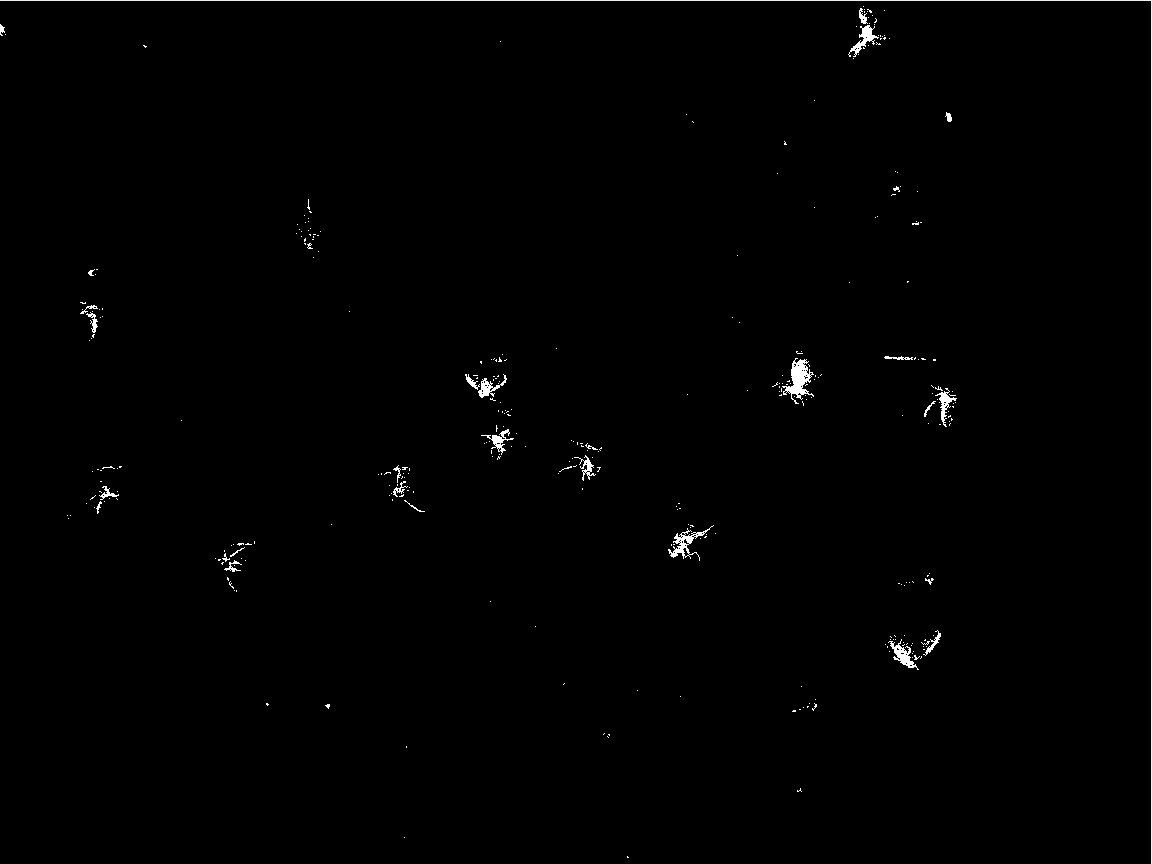

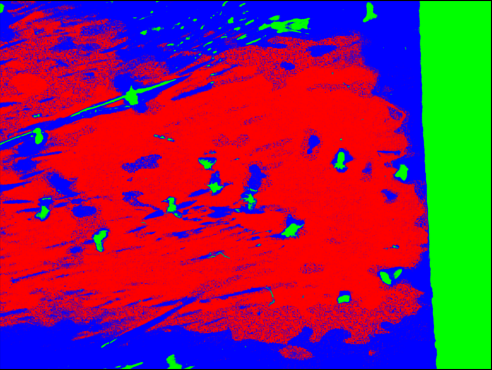


1. (d)


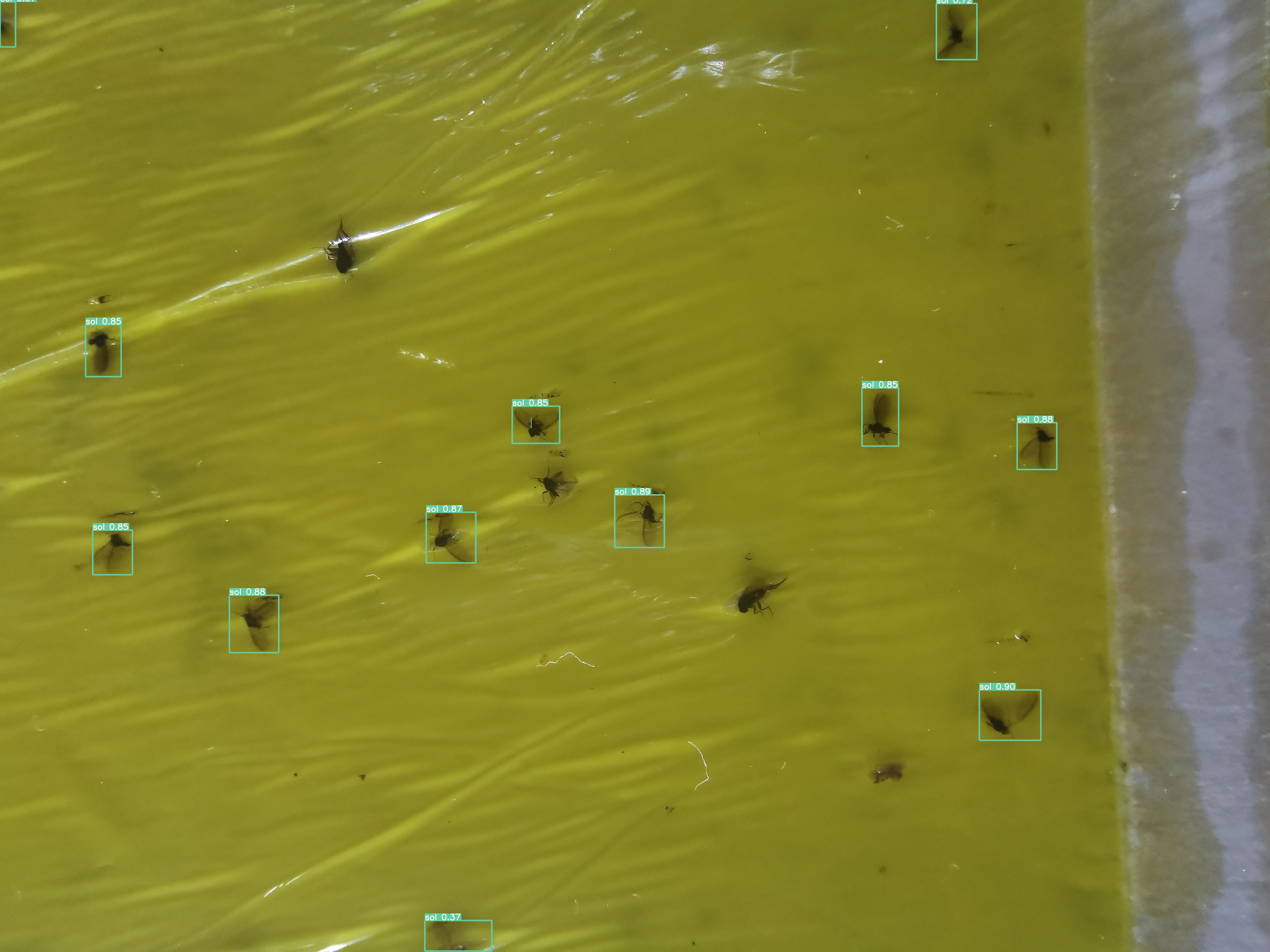


(e)

**Suplementary Figure 6**. Comparison of YOLO v5l model with machine learning methods. (a) Original image (b) Ground Truth image (c) Vision-based method (d) Gaussian Mixture Model (GMM) (e) YOLO v5l model.


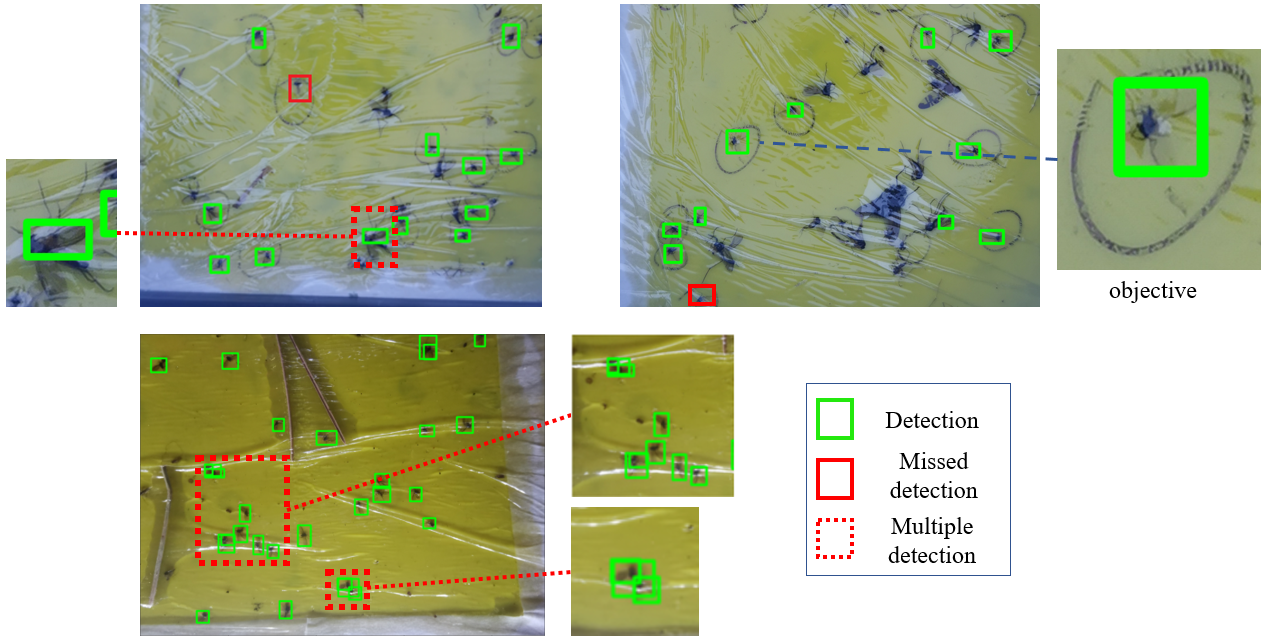


**Suplementary Figure 7.** Limitations in Detection results.


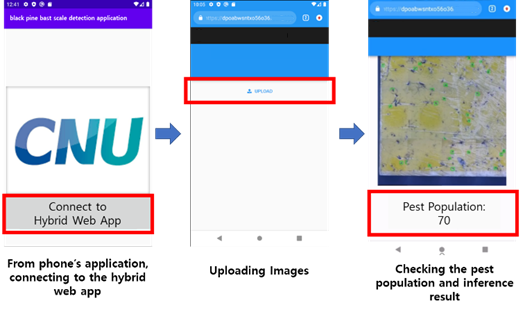


**Suplementary Figure 8.** Pest Black pine bast scale application framework.

**Suplementary Table 1.** Specifications of deep learning workstation.

| S.No. | Hardware / Software specifications | |
| --- | --- | --- |
| 1. | Processor | INTEL XEON GOLD 6230 PROCESSOR x 2 |
| 2. | Graphic Cards (GPU) | NVIDIA QUADRO RTX 4000x3 |
| 3. | RAM | 256 GB |
| 4. | Operating System | UBUNTU 18.04.3 LTS |
| 5. | Graphic Drivers | CUDA 10.1 & cuDNN 7.6.5 |
| 6. | Python Version | 3.6.8 |

**Suplementary Table 2.** Training duration of various YOLO models.

| Model | Training time (Hours) |
| --- | --- |
| YOLO v3 | 1.28 |
| YOLO v4 | 1.29 |
| YOLO v5s | 1.50 |
| YOLO v5m | 1.55 |
| YOLO v5l | 2.85 |
| YOLO v5x | 2.95 |

**Suplementary Table 3.** Comparison of YOLO v5l model with other models for pest detection.

| Model | Size (Mb) | Training time (hours) | Inference Time (sec/image) |
| --- | --- | --- | --- |
| Fast-RCNN | 401 | 8.21 | 0.098 |
| Faster-RCNN | 157 | 7.14 | 0.029 |
| RetinaNet | 218 | 7.36 | 0.054 |
| YOLO v5l | 89 | 2.85 | 0.018 |
